# Endoscopic management of duodenal perforations during endoscopic ultrasound: a case series

**DOI:** 10.3389/fmed.2026.1705286

**Published:** 2026-02-12

**Authors:** Chuangye Zhang, Shutian Zhang

**Affiliations:** 1 Department of Gastroenterology, Beijing Friendship Hospital, Capital Medical University, Beijing, China; 2 Department of Gastroenterology, Beijing Erlonglu Hospital, Beijing, China

**Keywords:** duodenal perforation, endoscopic ultrasound, over-the-scope clip (OTSC), surgical intervention, therapeutics

## Abstract

**Objective:**

The study aimed to investigate the feasibility and safety of using an over-the-scope clip (OTSC)for closing duodenal perforations caused by endoscopic ultrasound (EUS) procedures, as well as to evaluate the efficacy of definitive management strategies for duodenal perforations induced during EUS.

**Materials and methods:**

A retrospective analysis was conducted involving patients who developed iatrogenic duodenal perforations during linear EUS at the Digestive Endoscopy Center of Beijing Friendship Hospital, Capital Medical University, from January 2017 to December 2024. Diagnoses, management, and outcomes were identified and retrospectively reviewed in four of these patients.

**Results:**

Among 5,174 linear EUS procedures, four cases of perforation occurred (0.077%). All patients were women, with a mean age of 76 years (range: 71–81). Perforations were caused by the curvilinear probe in all cases: Three during diagnostic EUS procedures and one after EUS-guided fine-needle aspiration biopsy. Defect sizes ranged from 3 mm to 15 mm. All perforations with conservative endoscopic management were successfully closed without surgical intervention. The hospitalization duration varied widely, ranging from 32 to 92 days, due to differences in the clinical conditions of the inpatients.

**Conclusion:**

Endoscopic closure of iatrogenic duodenal perforations ≤ 15 mm using an OTSC is safe and effective when performed by experienced endoscopists. Early identification of perforation, guided by clinical symptoms and risk factors, followed by rapid evaluation and endoscopic intervention, may reduce complications and facilitate recovery.

## Introduction

Linear endoscopic ultrasound (EUS) is essential for diagnosing and sampling benign/malignant biliary-pancreatic diseases, pancreatic cystic lesions, and solid gastrointestinal tumors. A systematic review and meta-analysis of EUS-guided pancreatic biopsy (1) demonstrated that the incidence of complications is very low. While generally safe, complications such as perforation (0.03%) and infection (0–8%) may occur (2). With the increasing adoption of interventional EUS techniques, iatrogenic duodenal perforations may become more frequent. Historically, open surgery was considered the standard treatment for duodenal perforation. However, with continuous advancements in endoscopic closure techniques, accumulating evidence in recent years indicates that endoscopic closure may obviate the need for open surgical repair (3–5). This study evaluates the feasibility, efficacy, and safety of endoscopic closure for EUS-related duodenal perforations. Herein, we present a retrospective analysis of the feasibility, efficacy, and safety of EUS device-based repair for iatrogenic duodenal perforations caused by EUS procedures at our institution’s Digestive Endoscopy Center, along with a report of our experience.

## Materials and methods

### Study design

Retrospective analysis was conducted on iatrogenic duodenal perforations that occurred during linear EUS procedures from January 2017 to December 2024. All perforations were caused by a curvilinear probe EUS scope (Pentax-EG3270UK/EG-3870UTK; Pentax China, Beijing). Perforations resulting from duodenoscopy, EUS-guided interventions, or cystfenestration were excluded.

Perforations were classified according to the Stapfer system (6):

Type I: Duodenal wall perforation (due to excessive pressure).
Type II: Periampullary perforation (sphincterotomy-related).
Type III: Biliary or pancreatic duct perforation (instrumentation-related).
Type IV: Retroperitoneal air (due to over-insufflation).

### Data collection

Variables included age, sex, comorbidities, indication, procedure, perforation site/size, closure technique, year of occurrence, hospital stay, histopathology of the mass, CT/EUS findings, years of EUS practice, and trainee involvement (yes/no) (Tables 1, 2).

**Table 1:** Patient demographics and perforation characteristics.

Case	Age/Sex	Indication	Comorbidity	Perforation size (mm)	Perferation site	Closure technique	Trainee involvement
1	81/F	Duodenal papilla mass	HT^*^	12–15	Posterior bulb wall	OTSC* + TTSCs* + triple-lumen tube	No
2	71/F	Duodenal papilla mass	None	8–10	Opposite papilla	TTSCs + nylon loop triple-lumen tube	No
3	78/F	CBD dilation Pancreatic mass	HT	3–5	Descending duodenum	OTSC + TTSCs nasogastric tube	No
4	74/F	Descending lipoma	AF^*^	12–15	Descending duodenum	OTSC + TTSCs + triple-lumen tube	Yes

*TTSCs: Through-the-scope clips; HT: Hypertension; AF: Atrial fibrillation; CBD: Common bile duct.

OTSC: Over-the-scope clip CT: Computed Tomography; EUS: Endoscopic Ultrasound; PD: Pancreatic duct.

The table summarizes the clinical profiles and endoscopic management of the four female patients (mean age 76 years, range 71–81) with iatrogenic duodenal perforations following EUS. Perforation sizes ranged from 3 to 15 mm. Of them, three patients were diagnosed and treated endoscopically within 6 h. All defects were successfully closed using conservative endoscopic techniques—over-the-scope clips (OTSCs) with or without through-the-scope clips (TTSCs). Closure was supplemented in three cases with a triple-lumen feeding tube (retention 9–21 days), and in one case with a nasogastric tube. No surgical intervention was required, and trainee involvement was documented in one case.

**Table 2:** Clinical outcomes.

Year of occurrence	Hospital stay (d)	Discovery Time (h)	Histopathology of the mass	CT/EUS findings	Duration of triple-lumen trainee feeding tube placement (d)	Years of EUS practice
2018	34	Promptly	Mild epithelial dysplasia	Enlarged major duodenal papilla, common bile duct dilation	9	10
2021	32	Promptly	High-grade epithelial dysplasia	Duodenal adenoma	15	2
2021	92	72	No malignancy identified	Low-density nodule in the head of the pancreas, common bile duct dilation, pancreatic duct dilatation	21	5
2023	49	5	No	Duodenal lipoma	14	0.5

This case series describes four iatrogenic duodenal perforations occurring between 2018 and 2023. All perforations were detected during the procedure (within ≤6 h) except for one case with a 72-h diagnostic delay. The histopathological findings of the underlying lesions varied and included epithelial dysplasia (both mild and high-grade), a benign pancreatic nodule, and a duodenal lipoma. Preprocedural imaging (CT/EUS) consistently identified relevant abnormalities, such as duodenal papilla enlargement, bile duct dilation, and pancreatic lesions. The endoscopist’s experience at the time of the procedure ranged from 0.5 to 10 years. Post-management hospital stays varied considerably (range 32–92 days), with the longest stay corresponding to the case with delayed diagnosis.

Case 1: a periampullary lesion was initially suspected. CT imaging revealed only common bile duct dilation. Although an EUS examination of the ampulla and periampullary region was planned, a perforation occurred during the procedure.

**Figure 1:**
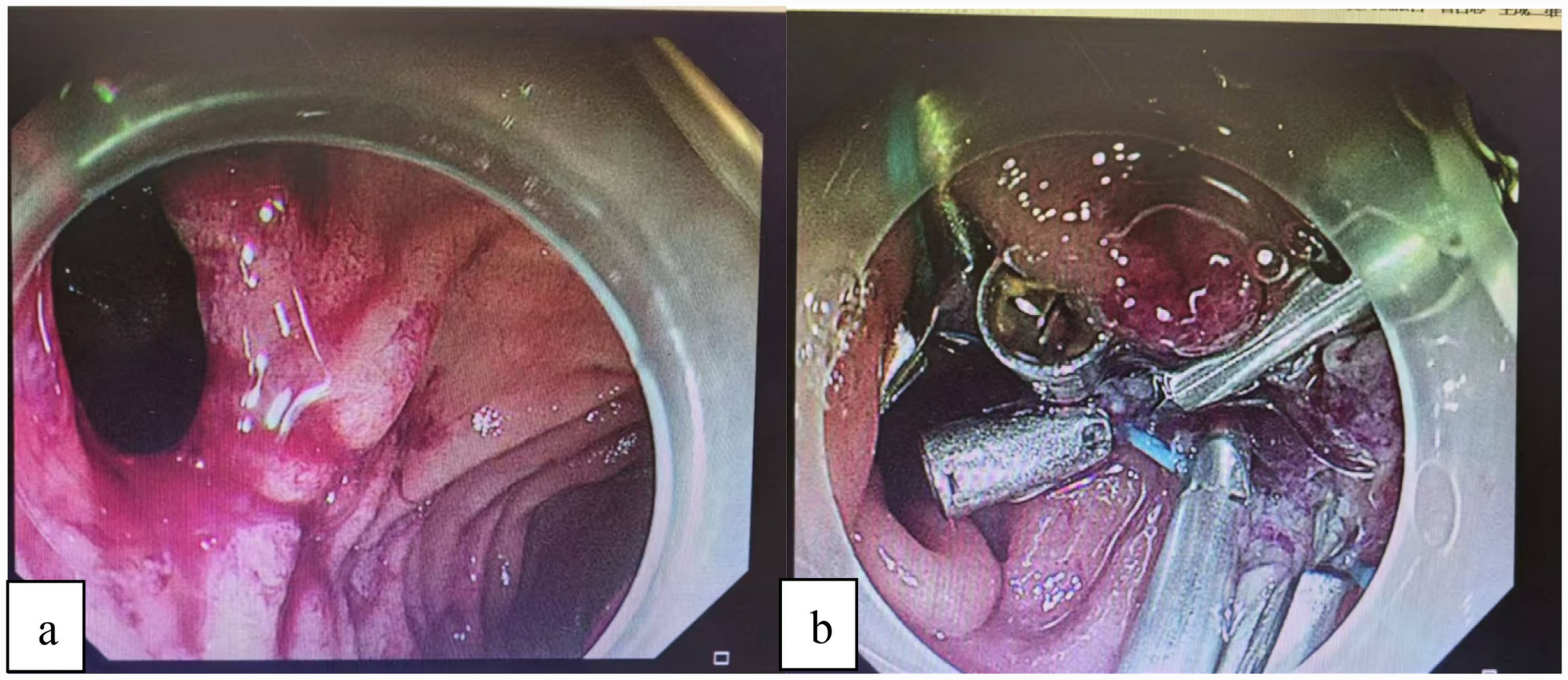
**(a)** Following an EUS examination, a perforation was identified in the posterior duodenal wall. We used a therapeutic endoscope (with a 3.2 mm channel) equipped with a transparent cap and connected to a carbon dioxide insufflation system. This provided optimal visualization and full exposure of the entire perforation site. **(b)** After multidisciplinary discussions with the anesthesia and surgical teams at the Digestive Endoscopy Center, we proceeded to directly use endoscopic clips to perform purse-string closure of the perforation.

Case 2: upper endoscopy showed a pedunculated polypoid lesion adjacent to the papilla, with biopsy confirming high-grade dysplasia. The CT scan was normal. During the scheduled EUS to assess the relationship of the lesion to the papilla, a perforation occurred on the contralateral side of the papilla (Figure 1).

Case 3: imaging findings and the clinical presentation upon admission suggested a lesion in the pancreatic head causing compression of the common bile duct and pancreatic duct, resulting in ductal dilation. To obtain a pathological diagnosis for further management, EUS-guided pancreatic puncture was performed as planned. Three hours post-procedure, the patient developed abdominal pain, and a duodenal perforation in the descending portion was confirmed 72 h later. The perforation was subsequently closed via endoscopic OTSC placement.

Case 4: upper endoscopy revealed a yellowish submucosal lesion in the descending duodenum, measuring approximately 2 cm in diameter. During the EUS procedure, the operator encountered poor visibility while navigating the scope through the junction of the duodenal bulb and the descending duodenum. Although the endoscopist proceeded to advance the scope into the descending duodenum, a thorough post-procedural inspection of the mucosa surrounding the suspected lipoma was not performed. Subsequently, a duodenal perforation in the descending segment was endoscopically confirmed 5 h later.

### Technical definitions

Technical success: secure closure using an over-the-scope clip (OTSC) or clips combined with nylon loops, with no leakage identified on contrast study.

Clinical Success: Absence of adverse events and CT-confirmed closure.

We primarily performed measurements under direct visualization using an Olympus therapeutic gastroscope (model GIF-Q260J) equipped with a transparent cap. The transparent cap (model D-201-11804) has an outer diameter of 11.8 mm and an inner diameter of 10.5 mm. It should be noted that excessive air insufflation was avoided during measurements. However, the aforementioned method has limitations, such as the influence of duodenal peristalsis and the extent of mucosal edema on the assessment of lesion margins.

### Endoscopic closure procedure

1. Upon confirming the diagnosis of duodenal perforation, the patient is typically transferred to an X-ray-equipped procedure room. Given the possibility of perforation, EUS-related procedures should be performed with CO_2_ insufflation to reduce the risk of pneumoperitoneum.
2. An Olympus therapeutic endoscope (with a 3.2-mm working channel) is then introduced, and an over-the-scope clip (OTSC, Ovesco Endoscopy GmbH, Tubingen, Germany) is deployed under endoscopic guidance to achieve immediate closure of the defect.
3. Under fluoroscopic guidance, a triple-lumen feeding tube is placed to provide postoperative enteral nutrition and gastrointestinal decompression.
4. The patient is kept fasting. Imipenem (Merck, Rahway, NJ, USA) 1.0 g is administered intravenously every 6 h to prevent infection, esomeprazole (AstraZeneca, S€odert€ alje, Sweden) is administered to inhibit gastric acid secretion, and somatostatin (Serono, Aubonne, Switzerland) is administered to inhibit pancreatic secretion. These treatments are continued for 1 week.
5. Following the diagnosis of duodenal perforation, the patient routinely receives a one-week comprehensive medical regimen consisting of fasting, intravenous fluid resuscitation, anti-infective therapy, acid suppression, and pancreatic enzyme inhibition. After treatment, if the patient shows complete resolution of abdominal pain and normalization of inflammatory laboratory markers, a small volume of liquid is administered via the jejunal port of the triple-lumen feeding tube under close monitoring for tolerance. Once adequate tolerance is achieved, feeding is gradually advanced to standardized enteral nutrition. Once enteral nutrition is well tolerated, the triple-lumen feeding tube is removed and oral intake is resumed.

### Ethical approval

This study was approved by the Institutional Review Board (2018-P2-191-01).

## Results

Among 5,174 linear EUS procedures, 4 cases of iatrogenic duodenal perforation occurred (incidence 0.077%). All patients were women, with a mean age of 76 years. The perforations resulted from the curvilinear probe during diagnostic EUS in three cases and from EUS-guided fine-needle aspiration in one case, with defect sizes ranging from 3 to 15 mm. All were successfully managed endoscopically without the need for surgical intervention or intensive care unit admission.

Diagnosis was based on progressive abdominal pain within 24 h post-procedure, localized peritoneal signs, elevated inflammatory markers, and confirmed free intraperitoneal air on computed tomography. Two cases were recognized intraprocedurally and closed within 3 h. The remaining two perforations, located in the descending duodenum, exhibited subtle initial symptoms, leading to diagnostic delays of 72 h and 5 h, respectively. Both cases required repeat imaging for confirmation and were ultimately closed endoscopically with adjunctive triple-lumen feeding tube placement. These cases underscore that perforations in the descending duodenum often present with attenuated clinical signs, necessitating heightened vigilance, serial monitoring, and timely re-imaging to prevent delayed intervention.

## Discussion

Gastrointestinal perforation during EUS is rare. Diagnostic endosonography has traditionally been associated with a very low rate of complications (1–2%) (7).

Therapeutic EUS procedures for various pancreatic and biliary indications show a high technical and clinical success rate, with a low incidence of adverse events (8). Consequently, available data suggest that the rate of perforation during EUS procedures is comparable to that of standard endoscopy. Although duodenal perforation during EUS procedures is very rare, it can have serious consequences if not recognized and managed with early and reliable closure (9). Perforation remains the most feared adverse event in endoscopy. Its management may require surgery, entails additional costs, and raises liability issues with potential legal implications (10). Next, based on our own case series, we analyze the causes or mechanisms of perforations induced by EUS focusing on three aspects.

**Figure 2:**
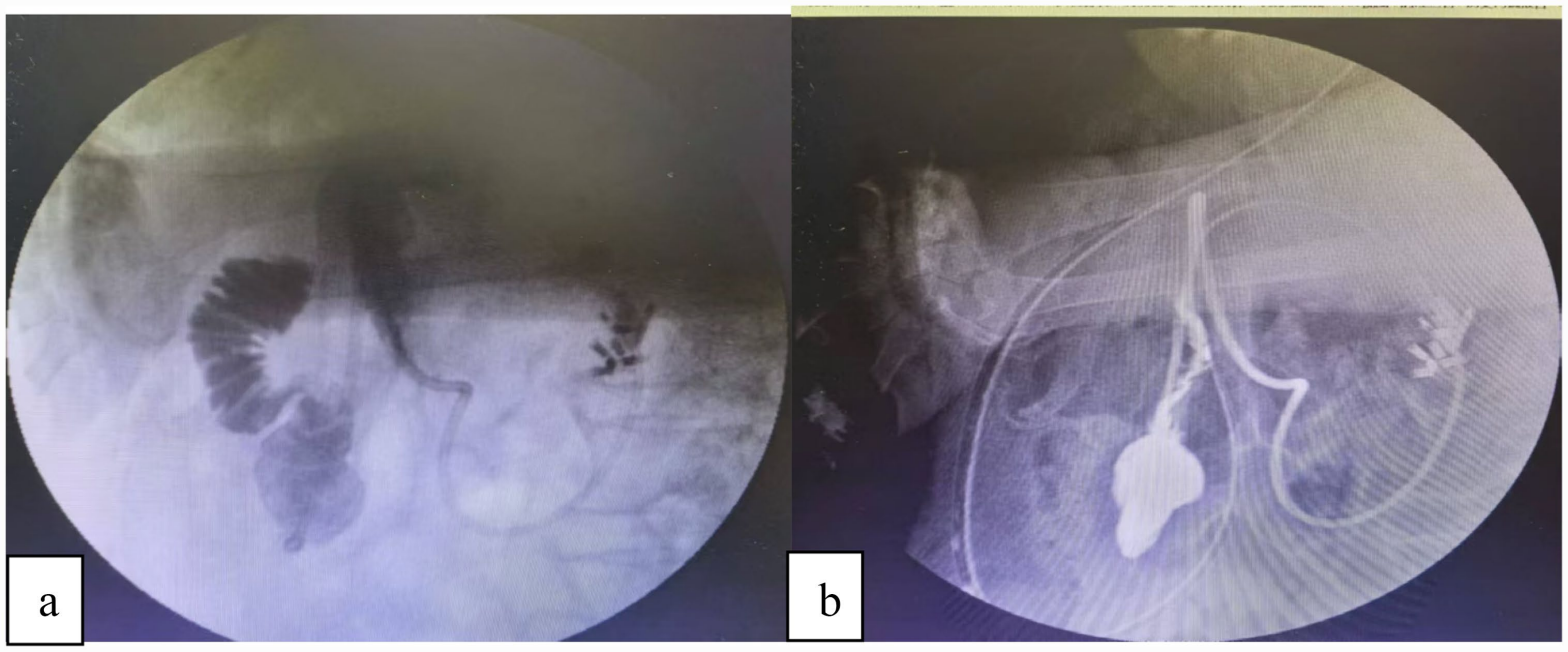
**(a)** The duodenal perforation was successfully closed endoscopically. Subsequently, under digital subtraction angiography (DSA) guidance, a guidewire was advanced through the endoscope’s working channel alongside a catheter, with its tip positioned in the jejunum. A triple-lumen feeding tube was then placed over the guidewire. **(b)** Contrast injection confirmed correct tube placement: The gastric port was located within the stomach, and the jejunal limb extended beyond the closure site. Furthermore, no evidence of contrast extravasation was observed.

First, a guidewire is introduced through the instrument channel of the transnasal gastroscope and advanced into the upper jejunum, approximately 20 cm distal to the ligament of Treitz. After confirming its position by injecting contrast medium, the gastroscope is withdrawn while the guidewire is left in place. Subsequently, the pre-lubricated triple-lumen feeding tube is advanced over the guidewire into the jejunum. The negative-pressure suction port is adjusted to the gastric antrum, and proper placement is reconfirmed by contrast injection. The guidewire is then carefully removed, and finally, the triple-lumen feeding tube is securely fixed to the cheek. This triple-lumen tube system provides integrated management through: (1) Direct decompression and anastomotic protection: Continuous low-pressure suction of gastroduodenal contents reduces intraluminal pressure and minimizes fluid exposure to the perforation site, creating a favorable environment for healing. (2) Early enteral nutrition: Distal jejunal feeding via a separate lumen maintains gut barrier function, prevents bacterial translocation, and supports tissue repair by providing essential protein and energy. (3) Pressure regulation and safety: The pressure-regulation lumen stabilizes suction pressure to prevent mucosal injury and maintains tube patency (Figure 2).

In certain cases, perforations may also result from anatomical variations or pathologies, such as an ulcer, tumor-induced compression, or inflammatory stenosis. Under these conditions, performing EUS and attempting to traverse the stricture may require complex maneuvers, which may place undue tension on the duodenal wall, potentially causing perforation with a risk of an extensive tear. In other cases, the risk of perforation may be related to the design of the EUS scope. Compared to standard gastroscopes (outerdiameter:9-11 mm,210°upward), EUS scopes (12.40–14.65 mm,130°upward) have a larger outer diameter, more limited upward angulation, reduced flexibility and maneuverability, and a longer rigid distal segment housing the ultrasonography transducer. In addition, the side-viewing optics further complicate maneuverability through the proximal duodenum. These factors collectively contribute to limited endoscopic visualization during advancement into the second portion of the duodenum, increasing the risk of mechanical trauma—particularly in areas with acute angulation. Endoscopists using side-viewing endoscopes, such as duodenoscopes, should enhance practical training, gain experience through systematic practice, and operate under the direct supervision of senior endoscopists. It is also essential to fully recognize and assess the risk of iatrogenic perforation associated with these procedures. Blind advancement or withdrawal of a side-viewing endoscope under conditions of insufficient visualization carries a risk of gastrointestinal perforation.

The management of EUS-related perforation in these four female patients underscores the significant challenges of endoscopic therapy. Below, we discuss these findings in two key contexts: Identification of risk factors and management strategies.

### Identification of risk factors

A patient’s unusual anatomy may increase the risk of perforation during traversal of the duodenal sweep with a stiff US transducer (11). The following factors have been independently associated with higher perforation rates during EUS (12–15): Trainee involvement, operator inexperience, advanced patient age, a history of difficult esophageal intubation, the presence of esophageal malignancy, and cervical spine osteophytes. Astonishingly, all perforation cases occurred in older women, which is consistent with previous literature (10). However, studies have shown EUS safety in older patients (16, 17), in short, endoscopists must be cognizant of these risk factors when performing EUS and counsel patients accordingly regarding the risk of perforation.

### Management strategies

Over the past 20 years, there have been numerous reports of EUS-related duodenal perforation (18, 19). However, there is no uniform standard for the treatment of EUS-related duodenal perforation. Iatrogenic perforations represent a life-threatening complication, for which surgical intervention was considered the standard treatment prior to 2014 (20). Linear EUS scopes share oblique-viewing optics with duodenoscopes, and their fundamental manipulation techniques are largely identical. Consequently, the assessment and management of duodenal perforations caused by linear EUS should follow the classic Stapfer classification system—the established standard for duodenoscope-related perforations (6).

**Figure 3:**
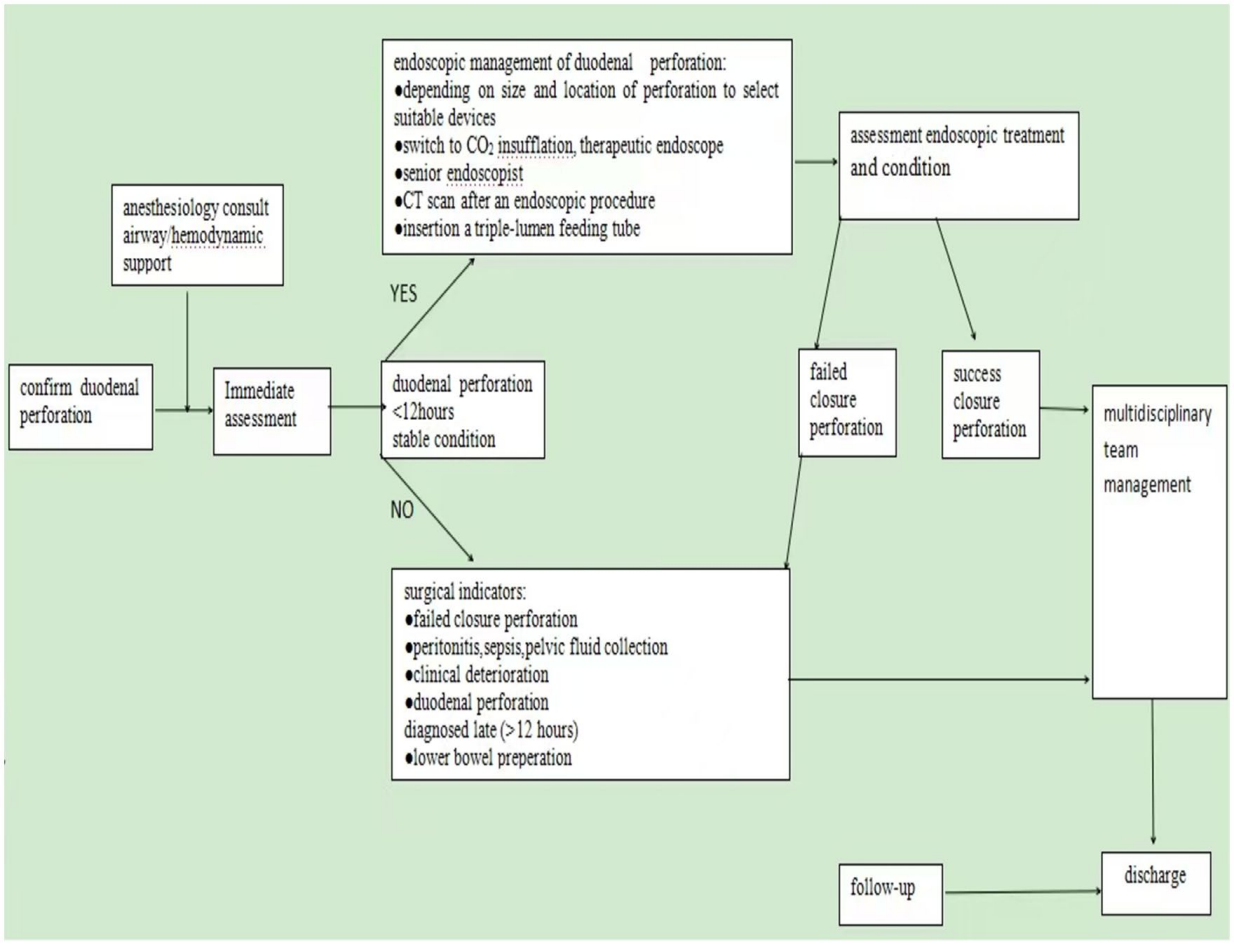
Endoscopic management strategies. Outlines endoscopic management strategies for duodenal perforations induced during EUS procedures. Endoscopic closure should be prioritized when a duodenal perforation is identified within 6 h in a clinically stable patient and an experienced endoscopist is available. Surgical intervention is recommended in cases of failed endoscopic closure or clinical deterioration.

Over the past 10 years, many scholars have advised that the choice of treatment should be based on the type of perforation (21, 22). It is generally accepted that type I perforations require prompt surgical intervention (3, 9). However, recent studies have recommended endoscopic treatments, including clipping, endoloop, and over-the-scope clip (OTSC), for type I perforations (23–26). The European Society of Gastrointestinal Endoscopy (ESGE) recommends endoscopic treatment of duodenal iatrogenic perforation if it is recognized immediately or early (<12 h) after the procedure. In the case of failed endoscopic treatment, the patient requires immediate surgery. If the duodenal iatrogenic perforation is diagnosed late (>12 h), surgical management is indicated in the case of contrast medium extravasation on the CT scan and/or deterioration of the patient’s condition. If the patient is clinically well, without contrast medium extravasation, the patient may be treated conservatively (27). In our four cases, three cases were diagnosed and managed conservatively within 6 h postoperatively. These patients underwent endoscopic therapy, including the placement of a triple-lumen feeding tube. They recovered well and were discharged without complications. In Case 3, the diagnosis was delayed until 72 h postoperatively. This patient’s postoperative hospital stay was three times longer compared to those who were diagnosed earlier and developed more complications, resulting in a slower recovery time. Iatrogenic perforation is optimally managed using a multidisciplinary strategy, involving the expertise of endoscopists, radiologists, and surgeons requiring immediate availability. Based on our experience with endoscopic management of duodenal perforation, the following management strategy is recommended when a duodenal perforation occurs during EUS procedures: (1) when it comes to an endoscopically identified perforation, the endoscopist should accurately document its size and location, with supporting images or videos. The duodenal bulb’s relative anatomical fixation facilitates early detection of perforations, as demonstrated in the first case of this study, where immediate identification occurred. However, its confined space restricts endoscopic instrument maneuverability, making complete defect closure with conventional through-the-scope clips (TTSCs) technically challenging—a finding consistent with existing literature (5, 10, 28). In contrast, perforations in the retroperitoneally situated descending and horizontal duodenal segments often present with subtle symptoms, resulting in delayed diagnosis. Their endoscopic accessibility is further limited, particularly in the horizontal segment, leading to prolonged procedural durations, as documented in prior studies (3, 29). The third case exemplifies this diagnostic challenge, with a descending duodenum perforation presenting atypically and being confirmed only after 72 h. Similarly, in the fourth case, the diagnosis was delayed until 5 h post-procedure. These observations align with established evidence that perforations in the descending duodenum are frequently associated with insidious clinical presentations and diagnostic delays. (2) For small perforations (<10 mm), through-the-scope clips (TTSCs) are generally recommended as the primary closure method. For larger defects (10–25 mm), over-the-scope clips (OTSCs) are preferred, either alone or in combination with through-the-scope clips or a nylon loop. (27). (3) We recommend that symptoms or signs suggestive of iatrogenic perforation following EUS should be promptly and thoroughly evaluated with computed tomography (CT) imaging. (4) We recommend that endoscopic closure should be considered based on the site, type, and size of the iatrogenic perforation, as well as the available endoscopic expertise at the treating center. It is recommended to switch to carbon dioxide (CO_2_) insufflation; a forward-viewing gastroscope with a 3.2-mm working channel should be used; and a triple-lumen feeding tube should be inserted to divert gastrointestinal contents, provide enteral nutrition, and decompress pneumoperitoneum. (5) We recommend that after endoscopic closure of a perforation, subsequent management decisions should be guided by the patient’s overall clinical condition. In cases of no or failed endoscopic closure of an iatrogenic perforation, and in patients whose clinical condition is deteriorating, surgical intervention is recommended (Figure 3).

### Limitations and future directions

The limitations of this study include its small sample size and single-center design. The small sample size (*n* = 4) restricts the generalizability and statistical power of the findings, making it difficult to accurately reflect the overall characteristics of EUS-related duodenal perforation in the broader population. Furthermore, prospective controlled studies must be conducted to optimize treatment strategies.

## Conclusion

Endoscopic closure of iatrogenic duodenal perforations ≤15 mm using an OTSC is safe and effective when performed by experienced endoscopists. Early recognition of perforation risk factors, timely and thorough assessment, adherence to standardized management protocols, and prompt endoscopic intervention can reduce complication rates and accelerate recovery.

## Data Availability

The original contributions presented in the study are included in the article/supplementary material, further inquiries can be directed to the corresponding author.
